# Measuring Mortality in Developing Countries

**DOI:** 10.1371/journal.pmed.0030056

**Published:** 2005-12-20

Some of the biggest public health successes in the Western world have come about because of simple records of people's deaths—their age at death, where they lived, and what they died of. Soaring lung cancer rates in the United Kingdom and United States around World War II, for example, led to life-saving research into the effects of smoking.

More than two-thirds of deaths worldwide are in developing countries, yet little is known about the causes of death in these nations. In India, for example, just one-third of deaths are registered, and of these, only one-third provide data on the cause of death. India's HIV/AIDS epidemic is rising—it may already have surpassed South Africa for the highest number of people infected. And like many other developing nations, the numbers of people dying from noncommunicable diseases such as heart disease and cancer are growing. Unlike most infectious diseases, the causes of noncommunicable ones can be the result of several risk factors, such as smoking, elevated blood pressure, or inherited genetic mutations.

With a population of 1 billion and growing, India urgently needs better data on the causes of death in its people if it is to take further steps to improve public health. To address this need, Prabhat Jha and colleagues designed a prospective study of 1 million deaths in India to run until 2014. They will monitor an expected 1 million deaths in nearly 14 million people across 2.4 million households to find patterns of disease according to gender, age, and region, and to better understand how risk factors such as tobacco and alcohol use and indoor air pollution are related to disease.

The study uses one of India's existing frameworks for measuring mortality, called the Sample Registration System (SRS). At present, the system covers 50,000 deaths every year. Two independent workers visit the households; one visiting every month and the other every six months. Their reports are collated and any discrepancies reconciled by a third person. To improve the system, Jha and colleagues are using an innovative method called a “verbal autopsy” to record details of death as reported by family or friends to a trained but nonmedical fieldworker. To ensure the robustness of the method, a random 10% of the fieldwork will be repeated by an independent audit team.

After validating the verbal autopsy method, the researchers began the first phase of the study, which ran from 1998 to 2003, and recorded deaths in 6.3 million people across 1.1 million urban and rural households nationwide. As of November 2005, the researchers have collected 140,000 verbal autopsy reports, and 35,000 have been coded and reconciled by two independent and trained physicians. They expect to record a total of about 300,000 deaths in the first phase and 700,000 in the second phase in 2004–2014, which will look at 7.6 million people in 1.3 million households.

Better knowledge of genetic risk factors—about which little is known in developing or developed countries—requires collection of biological samples. Jha and colleagues are also planning to test the feasibility of this by collecting dried blood spots or tubes of blood in SRS units in four to five Indian states.

Studying mortality in 14 million people is a huge challenge, but one that is necessary in view of India's vast population. As the researchers point out, direct measurement of the causes of death is a great deal more reliable than indirect estimation. By studying diseases that are common in one part of India but not in another, new risk factors should be discovered, and these are likely relevant worldwide. Mortality measurements will be key to the success of one of the world's largest public health initiatives—the Millennium Development Goals, which were set in 2000 when countries worldwide pledged to reduce by half or more the incidence of many diseases in poor countries. We will only know whether these goals have been met if we have reliable mortality statistics.

